# Notes and Notices

**Published:** 1883-05

**Authors:** 


### NOTES AND NOTICES.

The Minnesota State Homœopathic Society.—The seventeenth annual meeting will be held at St. Paul, May 15th, 1883. Officers: President, G. H. Hawes, M. D.; First Vice-President, D. M. Goodwin, M. D.; Second Vice-President, O. H. Hall, M. D.; Secretary and Treasurer, Arthur A. Camp, M. D.

Hahnemann's Medical Association of Louisiana.—The annual meeting of the Hahnemann Medical Association of Louisiana was held Friday evening, February 9th, at the Homœopathic Pharmacy, No. 130 Canal Street, and the following officers were duly elected to serve for the ensuing year: J. G. Belden, M. D., President; Walter Bailey, Jr., M. D., Vice-President; Mrs. Harriet C. Keatinge, M. D., Recording Secretary; Charles J. Lopez, M. D., Corresponding Secretary; Christian Sanders, M. D., Treasurer.

Gregg on Diphtheria.—Drs. A. Mattoli and G. Pompili have rendered their countrymen a service in translating into Italian Dr. R. R. Gregg's book on Diphtheria.

Hering's Guiding Symptoms.—We have recently received many inquiries as to this work. We therefore publish the following notice: Hering's *Guiding Symptoms,* volumes I, II, and III, are ready for delivery. Scale of prices: To stockholders, bound in cloth, $2.75 per vol.; in library leather, $3.25; in half morocco, $3.75; to non-stockholders, in cloth, $5.00; in library leather, $6; in half morocco, $7. Orders for stock or books and the remittances for the same may be sent direct to Messrs. J. M. Stoddart & Co., 1018 Chestnut Street, Philadelphia.

The "New Code" Again.—The New York allopathists are still quarreling over their new code. The opposing parties are organizing for the fight.

